# Changes in Heart Rate and Rhythm During a Crossover Study of Simulated Commercial Flight in Older and Vulnerable Participants

**DOI:** 10.3389/fphys.2019.01339

**Published:** 2019-10-24

**Authors:** Mark J. Meyer, Irina Mordukhovich, Gregory A. Wellenius, Murray A. Mittleman, John P. McCracken, Brent A. Coull, Eileen McNeely

**Affiliations:** ^1^Department of Mathematics and Statistics, Georgetown University, Washington, DC, United States; ^2^SHINE in the Department of Environmental Health, Harvard T.H. Chan School of Public Health, Boston, MA, United States; ^3^Center for Environmental Health and Technology, Brown University, Providence, RI, United States; ^4^Department of Epidemiology, Harvard T.H. Chan School of Public Health, Boston, MA, United States; ^5^Center for Health Studies, Universidad del Valle de Guatemala, Guatemala City, Guatemala; ^6^Department of Biostatistics, Harvard T.H. Chan School of Public Health, Boston, MA, United States

**Keywords:** aviation health, hypoxia, heart rate, heart rate variability, cardiovascular health

## Abstract

**Objectives:** Elderly passengers and those with preexisting disease are flying with increasing frequency and in-flight cardiac emergencies are a more frequent occurrence. We conducted a study of the physiological effects of simulated cabin altitudes and resulting lower oxygen levels among such passengers.

**Methods:** We monitored 41 participants in a hypobaric chamber for 2 days, one at an equivalent of 7,000 feet altitude (regulations limit pressurization to 8,000 feet) for a 4–5 h simulated flight and the other at ground level using generalized least squares models to account for repeated measures. We evaluated associations between simulated flight, heart rate (HR) and measures of heart rate variability(HRV) (root mean square of successive R-R interval differences [RMSSD], standard deviation of normal-to-normal intervals [SDNN], high-frequency power [HF], and low-frequency power [LF]).

**Results:** Heart rate was 3.9% (95% CI: 2.1, 5.8) higher on simulated flight days compared with non-flight days. The RMSSD was 10.6% (95% CI: −21.3, 0.05) lower during simulated flight days, indicative of reduced HRV. The remaining HRV measures were also lower on simulated flight days, though associations were less precise.

**Conclusion:** We report that typical simulated flight conditions elicit changes in cardiac autonomic control, suggesting sympathetic arousal or reductions in parasympathetic drive. Our findings, if confirmed, may suggest the need for guidelines to protect vulnerable passengers including prescreens, symptom evaluation after air travel, the use of oxygen concentrators, and education about healthy behaviors in flight.

## Introduction

Air transportation is a commonplace means of travel, with over 2.75 billion passengers flying on commercial airlines annually (Peterson et al., 2013). While flight is generally well tolerated, older flyers and those with cardiovascular disease (CVD) may be at risk of complications caused by reduced oxygen delivery in flight (Low and Chan, 2002). While commercial airplanes fly at altitudes of around 34,000 feet, Federal Aviation Administration (FAA) regulations limit cabin pressurization to an equivalent of 8,000 feet, which is the typical pressurization implemented by most aircraft. Pressurization to this equivalent is selected to balance preventing acute altitude-related health symptoms among flyers with operational demands on the aircraft. However, comprehensive acute and longer-term health effects of cabin pressurization have not been well characterized. Complications may be influenced by flight characteristics, activity during flight, lifestyle factors, and medical history, and include activation of the sympathetic nervous system, increased myocardial demand, paradoxical vasoconstriction, and alterations in cardiac autonomic control and hemodynamics that may in turn increase risk for flight-related cardiovascular events (Higgins et al., 2010; Wilkins et al., 2015).

The baby boomer generation (born between 1946 and 1964) are the first to fully utilize air travel and are expected to fly more than any previous generation of elderly passengers. With the projected tripling of the global population of people aged 60 and older by 2050, the number of elderly and unhealthy flight passengers will increase considerably (World Health Organization Media Center, 2004). Approximately 37% of the United States population has some form of CVD, including nearly 70% of those aged 60–79 years and over 80% of people aged 80 and older (Benjamin et al., 2018). Cardiac symptoms and events are estimated to cause 8% of in-flight health emergencies (Peterson et al., 2013). It is possible that cardiac effects may also occur in the days following a flight. With the advent of new fuel-efficient aircraft designs, ultra-long haul flights lasting upward of 19 h may expose vulnerable passengers to hypoxic environments for unprecedented durations.

Possible mechanisms underlying the potential risk of flight-induced cardiac events include changes in cardiac autonomic control due to, for example, changes in blood oxygen saturation or carbon dioxide levels that may lead to altered heart rate variability (HRV) and heart rate (HR). HRV reflects the variation of timing between heart beats and reflects autonomic modulation of the rhythmic activity of the sinus node (Task Force of the European Society of Cardiology and the North American Society of Pacing and Electrophysiology, 1996). Reduced HRV and increased HR are predictive of acute and chronic cardiovascular morbidity and mortality (Tsuji et al., 1996; Nolan et al., 1998; Perret-Guillaume et al., 2009). Flight, simulated flight, and flight-related environmental exposures are related to changes in HR and HRV as well as to cardiovascular events in previous studies (Jorna, 1993; Sauvet et al., 2009; Alvarez-Velasco et al., 2016). We hypothesized that we would observe associations between simulated flight conditions and unfavorable changes in HR and markers of HRV in a vulnerable population, and investigated this possibility in a crossover, single-blinded chamber exposure study.

## Materials and Methods

### Study Sample

Study inclusion/eligibility criteria were age over 50 years, recent air travel without health complications, and being currently well (i.e., no infections, fevers, etc.). We also selected a subgroup of stable heart disease patients to reflect the prevalence of this condition in aging passengers. We included current smokers because of the potential risks at altitude for these passengers (Nesthus and Wise, 1997). Our final sample was selected to reflect the aging population and included three main risk groups: (1) moderate cardiac disease patients, including coronary artery disease patients with a past history of severe blockage or infarction and congestive heart failure (CHF) patients classified as 1 and II according to New York Heart Association criteria [*n* = 13], (2) current tobacco smokers [*n* = 14], and (3) non-smokers without reported cardiac disease, but including those with a stable chronic disease diagnosis, such as hypertension, asthma, diabetes, obesity, or mental illness [*n* = 14]. Group membership was determined at the time of enrollment.

We recruited participants through clinics, newspapers, senior centers, and fitness centers in the Oklahoma City area, chosen because of proximity to the hypobaric chamber at the FAA Civil Aerospace Medical Institute (CAMI). Volunteers were accepted into the study on a rolling basis after a phone screen by a nurse practitioner that determined eligibility based on age, travel history, medical history, and availability to complete two study days in the chamber. Participants were scheduled for a clinical evaluation by a physician within 2 weeks if they met inclusion criteria. This exam excluded those with unstable health conditions and documented baseline oxygen saturation, pulmonary function, electrocardiogram readings, blood pressure, urine and blood tests, and body mass index. Out of the self-selecting group of volunteers responding to our study recruitment efforts, very few did not ultimately participate due to exclusion at either the phone screen or the medical examination, and of these, only one participant (a heart disease patient) did not complete the entirety of the study chamber because of a work conflict.

### Study Design

We monitored participants for 2 days in a hypobaric chamber, with a day off in between, during one of 7 weeks (December 2007-June 2008). We monitored all individuals included in the study (i.e., the sample or healthy older participants, including smokers and non-smokers, as well as the group of heart disease patients described above) before, during, and immediately after a 4 to 5-h flight simulation, once with pressurization equivalent to 7,000 feet altitude (below usual cruising altitudes of 8,000 ft.) and once with the chamber pressurized to near sea level altitudes (McNeely et al., 2011). Participants were blinded to the chamber exposure condition. Researchers were not blinded in case of potential health events. Exposure order was randomized by experimental group. Based on this, 30 participants received the flight condition on their first day in the chamber while 11 participants received the control condition first. Researchers provided meals and paid participants $300. All participants provided informed written consent in accordance with protocols approved by the Human Subjects Committees at the Harvard School of Public Health (Institutional Review Board Protocol #P15170-101), CAMI, and the University of Oklahoma Medical Center. This study was originally funded by the United States. FAA Office of Aerospace Medicine through the National Air Transportation Center of Excellence for Airliner Cabin Environment Research, with later additional funding from the United States National Institute of Health.

### Patient and Public Involvement

Patients were not involved in development of the research question and outcome measures, in the design of the study, or in the recruitment to and conduct of the study, except in so far as our research question was informed in part by medical events and symptoms that have occurred in-flight by passengers and flight crew members. Results from our study will be posted on our researchers’ website, www.fahealth.org.

### Instrumentation

The chamber was outfitted with twelve commercial airline seats arranged in four rows, which seated participants, a medical monitor, and a research assistant. Chamber gauges recorded humidity, temperature, noise, pressure, and carbon dioxide levels. Participants wore a LifeShirt^TM^ (Vivometrics, Inc., Ventura, CA, United States), a fitted vest made of lightweight Lycra material with embedded sensors, including a single-channel electrocardiograph. Data were recorded into an attached logger along with respiration measures, blood pressure, and pulse oximetry from sensors and peripheral devices, and were transmitted wirelessly to computer displays outside the chamber for monitoring cardiac waveforms in real time. ECG recordings were subsequently analyzed offline by trained technicians blind to exposures. We calculated time and frequency domain measures of HRV for 5-min intervals throughout the measurement period using standard software, including only normal sinus beats in the calculations and excluding any interval with >20% of the time spent in non-sinus rhythm (Goldberger et al., 2000).

We assessed the following frequency domain measures: high-frequency power (HF), a marker of parasympathetic drive, and low-frequency power (LF), a marker of both sympathetic and para-sympathetic activity (Shaffer and Ginsberg, 2017). We note that, while HF and LF are correlated with sympathetic and parasympathetic activity, they are measures of heart rhythm control rather than autonomic nervous system activity, which includes, for example, pupillary dilation salivary gland activity, gastrointestinal activity, electrodermal activity, and renal sympathetic activity (Wehrwein et al., 2016). We also assessed time domain measures of HRV: the standard deviation of normal-to-normal intervals (SDNN) and the root mean square of successive RR interval differences (RMSSD) (Shaffer and Ginsberg, 2017). SDNN is one of the most frequently assessed measures and reflects autonomic nervous system activity, whereas RMSSD reflects the beat-to-beat variance in HR and is related to vagally mediated changes in HRV (Ernst, 2017).

On both days, participants were instructed to behave as they would aboard a flight. They could eat, sleep, rest, read, watch movies, move about, or talk freely. The research assistant served meals and snacks. A bathroom was located in the back section. Phlebotomists, entering and leaving through an adjacent pressure-locked room, collected blood specimens for CAMI genomic studies.

### Statistical Analysis

We inspected data for missing values and normality and used median values derived from raw minute-level physiological data to trend 5-min averages of cardiac indices. We removed participants missing an entire day of observations from the analysis and ignored missing observations in analysis, leading to the removal of five participants: four heart failure patients and one non-smoker without cardiac disease. To ensure reliable HRV measurements, we retained observations with a ratio of NN to RR intervals greater than 75%, which excluded epochs with more noise and therefore less total information. Two participants, both smokers, were deemed to be outliers in their post minus pre-condition changes of RMSSD or SDNN, removal of these subjects did not affect our overall results (average differences for each participant shown in Supplementary Figure S1). For the primary analysis, measures of HRV were log-transformed to improve normality and stabilize the variance. The outcome of interest was percent change in HR/HRV, which was calculated as (e^b^-1)^∗^100%, where b is a coefficient from a longitudinal regression model; 95% confidence intervals (CIs) were calculated as (e^b±1.96*SE^-1)^∗^100%, where SE is the standard error associated with the regression coefficient (Park et al., 2005).

Trending the 5-min averages of RMSSD, SDNN, HR, HF, and LF resulted in one observation per HRV measure for every 5 min each participant was in the chamber. Thus, for each day in the chamber, a subject had at most 88 measurements available for analysis, with a few measurements taken before the start of the simulated flight or control—we refer to these as pre-condition measurements and measurements taken after the start of simulated flight or control as post-condition. To account for the crossover design, we conducted two difference-in-difference analyses—a statistical technique used to examine changes from baseline over time between two different treatments—comparing the change in HR/HRV from pre-condition to post-condition between the simulated flight and control days. We began with an unadjusted analysis of the average difference in pre-condition HR and HRV levels versus post-condition levels to visually demonstrate their raw changes due to altitude exposure. For interpretability, we perform this first analysis on the original, unlogged HR/HRV data. However, since the participants were repeatedly sampled on each day and also served as their own controls between days, the data is not independent. Thus, we use as our primary analysis an adjusted model to account for the repeated, time-varying structure of the data.

Using generalized least squares regression, a longitudinal regression method (Fitzmaurice et al., 2011), we controlled for within-participant variability by day via a 1st order auto-regressive [AR(1)] correlation structure placed on the participant-exposure level (Littell et al., 2000). In addition to fitting the difference-in-difference, we adjust this model for the time of day the participants enter the chamber as well as for the order in which participants received the treatment (order was block randomized). We do this to control for the effect of diurnal variation on HR and HRV and to account for a possible familiarity effect in case participants became used the chamber on the second day. Finally, we incorporate a participant-specific intercept to account for unmeasured confounding at the person-level. For participant i’s, jth measurement on the kth day in the chamber, denoted y_ijk_, our primary statistical model then has the form

log(y)ijk=b+0b+1ibE2xposure+ijkbC3onditionijk+bT4imeofDay+ijkbS5econdDayijk+bE6xposureCijk*ondition+ijkeijk

where Exposure_ijk_, Condition_ijk_, and Second Day_ijk_ are all indicator variables denoting, respectively, that the measurement came from the exposure day (Exposure_ijk_ = 1, 0 if control), was a post-condition measurement (Condition_ijk_ = 1, 0 if pre-condition), and came from the participant’s second day in the chamber (Second Day_ijk_ = 1, 0 if from first day). We assume normality of the error term, e_ijk_, and account for within-subject variability by placing an AR(1) correlation matrix for errors corresponding to participant i’s, kth day. Between-participant variability is accounted for by the variance of the e_ijk_ which we assume is not time-varying. For each log-transformed HRV and HR measure, we fit these models and compared associations between simulated flight and HR/HRV among all available participants as well as within the cardiac group, the non-smokers, and the smokers using percent change in y_ijk_ calculated as the difference between [exp(b_6_)-1]^∗^100% and [exp(b_3_)-1]^∗^100%. The variance for the difference in percent change was calculated using an application of the Delta Method (Casella and Berger, 2002).

To examine how time in the chamber modifies the observed associations between simulated exposure to cabin pressure and HR/HRV, we included an effect of duration of exposure interacted with the exposure. We also used a GLS models with AR(1) correlation structure to estimate effects for each outcome among the full study sample. As before, all duration models were adjusted for person-level effects, diurnal variation, and chamber effect. The statistical model used to assess the effect of duration is then

log(y)ijk=b+0b+1ibC2ondition+ijkbT3imeofDayijk+bS4econdDay+ijkbD5urationijk+bE6xposureCijk*onditionijk+bD7urationEijk*xposure+ijkeijk

where Duration_ijk_ measures the duration of time in the chamber for participant i, to measurement j, on day k. Duration starts at zero for all participants and is used to estimate a time-varying association between exposure and HR/HRV. The remaining model components are as defined above. All estimates for both this model and the primary model were generated using the gls function from the nlme package version 3.1-137 in R Version 3.5.0 (Pinheiro et al., 2018; R Core Team, 2018). The code used to perform these analyses is publicly available at https://github.com/markjmeyer/ChamberStudy, with data made available from the corresponding author upon request.

## Results

**FIGURE 1:**
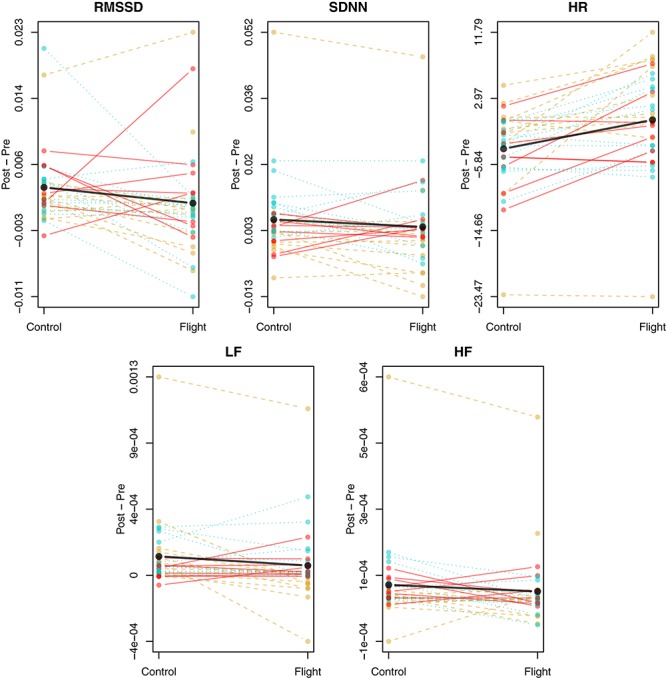
Unadjusted, average difference between pre-condition and post for heart rate (HR) and HRV (RMSSD, SDNN, HF, and LF) broken down by simulated flight and control day for each subject. The differences are first averaged over all available measurements for each subject’s control and flight days. Solid red lines and points denote each Heart Failure participant’s mean difference for a given day, dashed gold lines and points denote Healthy participants, and dotted cyan lines and points denote the Smokers. Solid black lines and points denote the mean value across all differences on the given day.

Table 1 describes participant characteristics. Participants had a mean age of 63 years, mean BMI of 28 kg/m^2^, 76% were white, and 13% were female. Most were current or previous smokers. Average unadjusted changes in pre- versus post-simulated flight HR and HRV levels (un-logged) for the flight and control days are shown in Figure 1 overall and for each participant individually. The change in HRV from pre-condition tended to be lower during simulated flight, whereas change in HR from pre-condition was higher during simulated flight. We present post-condition averages for both flight and control in the Supplementary Figure S2.

**TABLE 1:** Chamber study participant characteristics of those included in the final analysis^a^.

	**All *N* = 34**	**Healthy *N* = 13**	**Heart failure *N* = 9**	**Smoker *N* = 12**
Female	13(38%)	6(46%)	2(22%)	5(42%)
Age (years)	63.22(56.96,69.92)	66.59(61.34,70.44)	62.47(55.49,69.92)	60.88(52.16,67.12)
Age 65 and older	13(38%)	6(46%)	4(44%)	3(25%)
White	26(76%)	12(92%)	7(78%)	7(58%)
Ever smoker	27(79%)	7(54%)	8(89%)	12(100%)
Body mass index	28.37(24.20,31.40)	27.48(25.50,29.70)	31.28(30.70,34.30)	27.14(23.00,31.40)
Diabetes	7(21%)	0(0%)	7(78%)	0(0%)
Asthma	3(9%)	1(8%)	1(11%)	1(8%)
COPD	3(9%)	1(8%)	2(22%)	0(0%)
Baseline SaO2^∗^ over 95	29(85%)	9(69%)	8(89%)	12(100%)
Beta-blocker use	13(38%)	2(15%)	8(89%)	3(25%)

*^∗^SaO2, oxygen saturation. ^*a*^Descriptive statistics are presented overall and by subgroups of interest as frequencies and percentages or means and first and third quartiles.*

We found that the change in HR from pre-condition was 3.9% (95% C.I. [2.1, 5.8]) higher on the day of the simulated flight than on the control day (Table 2). We also found that the change in RMSSD from pre-condition was 10.6% (95% C.I. [−21.3, 0.05]) lower on simulated flight days. The remaining changes in HRV measures from pre-condition were lower on flight days: by 1.0% (95% C.I. [−12.3, 10.2]), 17.3% (95% C.I. [−42.0, 7.4]), and 11.5% (95% C.I. [−40.5, 17.4]) for SDNN, HF, and LF, respectively. However, these associations did not rise to the level of statistical significance (Table 2). We observed little evidence of variation by subgroup and thus do not report these results here.

**TABLE 2:** Adjusted relative difference in pre-condition versus post change in heart rate (HR) and heart rate variability (HRV) between control and flight.

**Outcome**	***N***	**Control^∗^**	**Flight^∗^**	**Relative difference (95% C.I.)**	***p*-value**
HR	34	−3.6%	0.3%	3.9 (2.1, 5.8)	<0.0001
RMSSD	34	13.2%	2.6%	−10.6 (−21.3, 0.05)	0.0511
SDNN	34	13.3%	12.3%	−1.0 (−12.3, 10.2)	0.8576
HF	34	24.9%	7.6%	−17.3 (−42.0, 7.4)	0.1691
LF	34	50.2%	38.7%	−11.5 (−40.5, 17.4)	0.4351

*HF, high-frequency power; LF, low-frequency power; RMSSD, root mean square of successive R-R interval differences; SDNN, the standard deviation of normal-to-normal intervals. ^∗^Estimates represent the percent change in outcome from pre-condition to post-condition. Relative difference is the difference between those two values, flight – control. Associations are adjusted for diurnal variation, chamber effect, and a fixed effect for each participant.*

**FIGURE 2:**
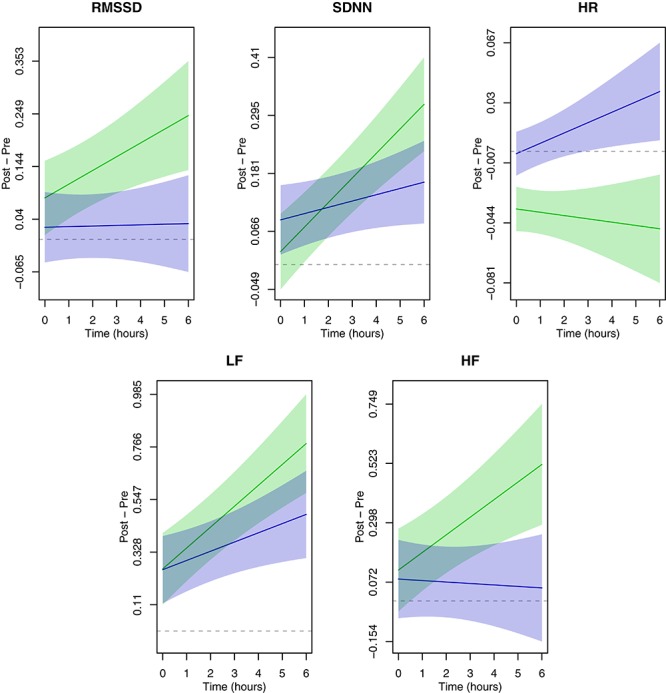
Time-varying associations between simulated flight exposure and heart rate (HR) and HRV (RMSSD, SDNN, HF, and LF). The simulated flight day is in blue; the control day is in green. Solid color lines indicate the average effect of exposure over time while the shaded bands are 95% confidence intervals for the corresponding line. Dashed gray lines are placed at zero for reference. Plotted estimates represent the changes from pre-condition for log(HR) and log(HRV) as a function of duration.

Time-varying associations between simulated flight exposure, HRV and HR are presented in Figure 2. The change in HR from pre-condition showed the largest difference between control and flight days, beginning with the start of the experimental condition and increasing as time went on. Changes in both RMSSD and HF from pre-condition had overlapping confidence bands at the beginning of monitoring, with separation occurring after 3–4 h of exposure for RMSSD and 4–5 h for HF. Hence, the longer participants were exposed, the greater the difference between control and flight days. SDNN and LF also showed an increasing difference between flight and control days with greater time in the chamber, though the results were less precise. We present individual and mean trajectory plots for all measures in the Supplementary Figure S3.

## Discussion

We report associations between simulated flight, higher HR, and lower HRV in a controlled exposure study among older, vulnerable passengers. Our results suggest that older passengers may show signs of cardiac autonomic stress at 7,000 feet equivalent cabin pressures, which are closer to sea level than the regulated limit of 8,000 feet. Reduced HRV and increased HR, even when short-term or occurring intermittently, can be predictive of cardiovascular morbidity and mortality and may represent mechanisms through which environmental conditions affect cardiovascular risk (Nolan et al., 1998; Neubauer, 2001; La Rovere et al., 2003; Perret-Guillaume et al., 2009). To our knowledge, we have conducted the first single-blinded study of the physiological effects of simulated flight in older passengers.

Several of the markers that we tracked suggest that flying may present more cardiovascular stress than previously understood. Cardiac sympathetic stimulation was apparent by an increase in HR and reduction in HRV over a 5-h simulated flight, which may be reflective of hypoxia-related changes in both physical and mental factors, as reduced oxygenation affects mental state and psychological factors such as stress are related to changes HRV (Vaernes et al., 1984; Kim et al., 2018). Homeostasis gave way after 3.5 to 4 h in our study, showing the importance of studying longer flight durations. Notably, most of the participants in our study would have been considered low risk for hypoxia according to their sea level oxygen saturations see Supplementary Figure S4 for average oxygen saturation and carbon dioxide levels post-condition on each day).

Prior studies evaluating changes in physiologic biomarkers at cabin altitudes report mixed findings (Akero et al., 2005; Bouak et al., 2018). Many studies were limited to HR only and the evaluation of in-flight hypoxia has been limited to either young, healthy passengers or very sick (usually pulmonary) patients. One study among chronic obstructive pulmonary disease (COPD) patients found evidence of increased HR and hyperventilation after 4 h of flying (Akero et al., 2005). These results are in line with our findings and argue that older and vulnerable passengers may be undergoing physiological stress. Our results are also consistent with findings of increased HR and reduced HRV among young, healthy men following a 1-h flight simulation (Oliveira-Silva et al., 2016). What this means for longer flights and in-flight or post-flight clinical outcomes is still to be determined, but our data suggest sympathetic arousal or reductions in parasympathetic drive (Silverman and Gendreau, 2009). We note that a secondary study conducted among our participants (using blood samples collected by study personnel during the single-blinded flight simulations in the hypobaric chamber described in this manuscript) reported the discovery of 155 genes involved in physiological responses to high altitude as well as alterations in pulmonary vasculature gene expression during simulated flight (Inimary et al., 2011). Previous studies have also reported health outcomes in the 48-h after flight and report outcomes, such as exacerbation of symptoms among COPD patients (Edvardsen et al., 2011).

Our results contribute to a more informed understanding about how older age affects responses at cabin altitudes, which is important given the increasing numbers of vulnerable passengers and the aging workforce of cabin crew and pilots. The average age for flight crew is 45 years, and mandatory retirement for pilots increased to 65 from 60 in 2007. Aging flyers may be particularly vulnerable to health effects from hypoxic environments because of anatomic, physiological and immunological changes related to aging, including alterations in structure and function of the heart and vasculature, blunted beta-adrenoceptor responsiveness, impaired autonomic reflex control of HR, and declines in other systems (e.g., pulmonary gas exchange and renal capacity) (Priebe, 2000; Sharma and Goodwin, 2006). Aging crew may be disproportionately affected by flight (relative to passengers) due to the frequency and duration of their exposures, increased metabolic demands during flight, and concomitant occupational problems such as sleep deprivation or exposure to ozone and noise (Griffiths and Powell, 2012). We recommend that future research evaluates physiological responses of aging flight crew members. Their health is highly understudied, yet they are responsible to protect passenger safety.

Many aging Americans are or have been exposed to tobacco smoke. A 2004 United States Surgeon General’s report on health effects of active and passive smoking found sufficient evidence to infer causal relationships with sub-clinical atherosclerosis, coronary heart disease, stroke, abdominal aortic aneurysm, and chronic obstructive pulmonary disease (COPD) (U.S. Department of Health and Human Services, 2004). COPD affects 14 million American adults, with 29 million showing evidence of clinical or subclinical impaired lung function, indicating a potential underdiagnosis (Rosenberg et al., 2015). A previous study among our participants found that many passengers with normal baseline blood oxygenation dropped to hypoxic levels during simulated flight, indicating that current medical guidelines deeming sea-level oxygenation greater than 95% as protective against the need for in-flight supplemental oxygen may underestimate the true risk of hypoxia for older flyers (McNeely et al., 2011). Similar findings of in-flight hypoxia were reported in a study of younger, healthy flyers (Winck et al., 2008). Notably, intermittent hypoxia is an effective stimulus for triggering cardiovascular events (Neubauer, 2001; Winck et al., 2008).

Individuals with lung or heart disease face special challenges in even mildly hypoxic environments (Rodenberg, 1988). Yet, aircraft are pressurized in accordance with research findings among healthy, fit, and younger participants. Further, we know very little regarding the potential health effects of increasingly common long-haul and ultra-long-haul flights of up to 19 h. The Aerospace Medical Association has released a position paper recommending research into the effects of mild hypoxia on passengers and crew (Aerospace Medical Association et al., 2008).

Our findings, if confirmed, may have implications for aircraft design features. The airline industry is studying how to best accommodate the needs and changing capacities of aging passengers (Ehrenman, 2005). Some designs, such as the Boeing Dreamliner, have already incorporated lower cabin pressures to minimize risks to passengers and crew, but these aircraft represent a very small portion of the current fleet.

Recent publications have addressed the concern for medical emergencies in flight, some of which argue a rise in emergencies (Peterson et al., 2013; Nable et al., 2015). Meanwhile, medical guidelines for passengers and crew have not been updated in many years, despite the changing demographics and risk profiles of passengers as well as changes in the commercial fleet. Such medical guidelines should also be disseminated more consistently to primary care providers and specialists (especially cardiologists and pulmonologists). Further, clinicians should counsel passengers and crew members regarding self-protective behaviors, including hydration, judicious use of alcohol, sleeping medications, and sedatives, movement during flight to increase circulation, and adequate rest before, during, and after flight.

Federal Aviation Administration recommendations for medical emergency kits have not been updated in over 15 years, and there are no current recommendations for stocking pulse oximeters or supplemental oxygen (Verjee et al., 2018). We note that new devices allow for flyers to easily take their own blood oxygenation readings, thereby making needed information on reactions to flight readily available.

The changes in HR and HRV that we observed in this study do not rise to a level of clinical concern. However, shifting the distribution of HR and HRV measures may be of public health concern because of changes among “borderline” individuals. Pressurization levels and flight durations in our study represent a lower exposure than many flyers would receive given standard pressurization to 8,000 feet and long flight durations. Intercontinental flights from the United States are at least twice the length of our simulation. In addition, concomitant flight exposures such as noise and circadian disruption were not simulated in our study and could also affect HR or HRV. Similarly, we were not able to control for other personal factors and measures that could influence HRV on control and simulated flight days, including differences in respiration, muscle contraction, movement, and psychological and cognitive factors, which could potentially systematically differ between experimental conditions according to levels of oxygenation. However, our crossover design involved measuring responses within the same participants, thereby decreasing person-level variability, and the main purpose of our study was to investigate changes in HRV in relation to differing pressurization levels. The changes in HR and HRV we observed are notable regardless of the mechanism through which these measures are affected. For example, HRV may change due to a combination of altered cognition, respiration, and movement patterns due to oxygenation during a simulated flight.

In addition, while some of our participants were heart disease patients taking medications including beta blockers, which depress compensatory cardiac stimulation, the role of prescribed medications in passenger responses to flight was not investigated due to insufficient statistical power to evaluate interactions. The role of obesity or overweight in physiological responses to flight could not be thoroughly evaluated for the same reason. However, while our results were imprecise, we did not observe differences in exposure-outcome associations in preliminary analyses stratifying by beta blocker use or overweight status. We only collected information regarding beta blocker use, and not regarding other medications, as beta blockers were considered to be the most important medications with regard to cardiac autonomic control (Aronson and Burger, 2001).

We also note that participants’ usual travel patterns are relevant to the interpretation of our results due to potential adaptation responses to frequent flights, or conversely because frequent recent air travel may present a preexisting source of cardiovascular stress. However, we recruited elderly participants into our study, many of whom had heart disease, and none of our participants traveled frequently, nor was there a substantial range in frequency of flight among our study participants. Most concerning in our study was that several participants had no knowledge of a history of cardiopulmonary conditions, yet pulmonary function testing and electrocardiogram readings showed evidence of underlying disease. This underscores the reality that passengers may overestimate their fitness for flying, and so too may their care providers. Finally, part of our goal was to identify mechanisms mediating the association between hypoxic aircraft conditions and cardiovascular outcomes, and these results, if confirmed, suggest that effects on HR and HRV may be part of that pathway.

We report that typical flight conditions produced compensatory mechanisms in older passengers. Our study was innovative in that we collected continuous repeated measures using state of the art flight simulation and medical sensor technology. However, more work needs to be done. Our study was limited by a modest sample size and associated statistical power. Future studies should evaluate a broader range of markers, including oxygen desaturation and respiration, should include a larger number of participants (which would allow investigating associations stratified by smoking history, medical history, and other personal factors), and should evaluate associations with longer flight durations and pressurizations to higher altitude equivalents. While our focus was on older flyers, additional studies should be conducted among younger participants to better understand responses to flight within this portion of the population, as the generalizability of our findings to a younger and healthier population is unclear.

Learning about responses in flight is critical given the confluence of many cardiac stressors, including increasing numbers of aging or unhealthy flyers, longer and more crowded flights, repeated and prolonged exposures among flight crew and frequent flyers, circadian disruption, noise, possible chemical exposures, and the psychological stress of flying. With the advent of wearable technology, in-flight monitoring may be conducted through apps and sensors. Future studies should evaluate associations between flight, arrhythmias/electrophysiology, and blood biomarkers, such as cardiac creatine kinase, cortisol, and antioxidant and pro-oxidant enzymes.

The associations we report between simulated flight, higher HR, and lower HRV in a controlled study among older, vulnerable passengers suggest that older passengers may show signs of significant cardiac autonomic stress at flight altitudes. This is concerning because reduced HRV and increased HR, even when short-term or occurring intermittently, can be predictive of cardiovascular morbidity and mortality (Nolan et al., 1998; Neubauer, 2001; La Rovere et al., 2003; Perret-Guillaume et al., 2009). Such information is relevant when considering medical guidelines and in preparation for flight, for which protective measures may be taken. Pre-flight screenings and medical consultations among the elderly or vulnerable should also be more routine (Low and Chan, 2002). Our findings will guide future research into the health effects of flight, in particular regarding the risk of cardiovascular symptoms and events in flight.

## Data Availability Statement

The datasets generated for this study are available on request to the corresponding author.

## Ethics Statement

The studies involving human participants were reviewed and approved by the Human Subjects Committees at the Harvard School of Public Health, Civil Aerospace Medical Institute, and the University of Oklahoma Medical Center. The patients/participants provided their written informed consent to participate in this study.

## Author Contributions

MJM designed and conducted the statistical analyses for the manuscript, interpreted findings, and wrote the manuscript. IM wrote the manuscript and interpreted the study findings. GW designed the protocols and analyses for this study, contributed to interpretation of the findings and writing the manuscript, and provided expertise regarding epidemiologic methodology. MAM contributed to the study design, interpretation, writing the manuscript, and contributed clinical expertise regarding epidemiologic methodology. JM was responsible for the data management, processing, and statistical analyses. BC contributed to the study design and interpretation, and contributed expertise regarding biostatistics and epidemiologic methodology. EM initiated this study and was instrumental to its design, including research protocols and statistical analyses, as well as in interpreting study findings and writing the manuscript. All authors reviewed the manuscript prior to submission.

## Conflict of Interest

The authors declare that the research was conducted in the absence of any commercial or financial relationships that could be construed as a potential conflict of interest.

## References

[B1] Aerospace Medical Association, Aviation Safety Committee and Civil Aviation Subcommittee (2008). Cabin cruising altitudes for regular transport aircraft (Position Paper). *Aviat. Space Environ. Med.* 79 433–439. 10.3357/asem.2272.2008 18457303

[B2] Akero A. Christensen C. Advardse A. Skjonesberg O. H. (2005). Hypoxaemmia in chronic obstructive pulmonary disease patients during a commercial flight. *Eur. Respir. J.* 25 725–730. 10.1183/09031936.05.00093104 15802350

[B3] Alvarez-Velasco R. Masiuan J. DeFelipe A. Corral I. Estevez-Fraga C. Crespo L. (2016). Stroke in commercial flights. *Stroke* 47 1117–1119. 10.1161/STROKEAHA.115.012637 26892280

[B4] Aronson D. Burger A. J. (2001). Effect of beta-blockade on autonomic modulation of heart rate and neurohormonal profile in decompensated heart failure. *Ann. Noninvasive Electrocardiol.* 6 98–106. 10.1111/j.1542-474x.2001.tb00093.x 11333166 PMC7027697

[B5] Benjamin E. J. Virani S. S. Callaway C. W. Chamberlain A. M. Chang A. R. Cheng S. (2018). Heart disease and stroke statistics – 2018 update: a report from the American Heart Association. *Circulation* 137 e67–e492. 29386200 10.1161/CIR.0000000000000558

[B6] Bouak F. Vartanian O. Hofer K. Cheung B. (2018). Acute mild hypoxic hypoxia effects on cognitive and simulated aircraft pilot performance. *Aerosp. Med. Hum. Perform.* 89 526–535. 10.3357/AMHP.5022.2018 29789086

[B7] Casella G. Berger R. L. (2002). *Statistical Inference*, Vol. 6 Belmont, CA: Duxbury, 160–169.

[B8] Edvardsen A. Akero A. Hardie J. Ryg M. Eagan T. M. Skonsberg O. H. (2011). High prevalence of respiratory symptoms during air travel in patients with COPD. *Respir. Med.* 105 50–56. 10.1016/j.rmed.2010.10.006 20974527

[B9] Ehrenman G. (2005). Fly the grayer skies: as Baby Boomers reach senior citizenhood, Boeing looks for ways to meet their changing needs. *Mechanical Engineering-CIME* 127 SS8+. Gale Academic Onefile.

[B10] Ernst G. (2017). Hidden signals – The history and methods of heart rate variability. *Front. Public Health* 5:265. 10.3389/fpubh.2017.00265 29085816 PMC5649208

[B11] Fitzmaurice G. M. Laird N. M. Ware J. H. (2011). *Applied Longitudinal Analysis.* Hoboken, NJ: John Wiley & Sons, Inc.

[B12] Goldberger A. L. Amaral L. A. N. Glass L. Hausdorff J. M. Ivanov P. C. Mark R. G. (2000). PhysioBank, physiotoolkit, and physionet: components of a new research resource for complex physiologic signals. *Circulation* 101 E215–E220. 10851218 10.1161/01.cir.101.23.e215

[B13] Griffiths R. F. Powell D. M. (2012). The occupational health and safety of flight attendants. *Aviat. Space Environ. Med.* 83 514–221. 22606869 10.3357/asem.3186.2012

[B14] Higgins J. P. Tuttle T. Higgins J. A. (2010). Altitude and the heart: is going high safe for your cardiac patient? *Am. Heart J.* 159 25–32. 10.1016/j.ahj.2009.10.028 20102863

[B15] Inimary T. Burian D. McNeely E. (2011). Effects of hypoxia on gene expression in the elderly. *FASEB J.* 25:861.

[B16] Jorna P. G. (1993). Heart rate and workload variations in actual and simulated flight. *Ergonomics* 36 1043–1054. 10.1080/00140139308967976 8404833

[B17] Kim H. G. Cheon E. J. Bai D. S. Lee Y. H. Koo B. H. (2018). Stress and heart rate variability: a meta-analysis and review of the literature. *Psychiatry Investig.* 15 235–245. 10.30773/pi.2017.08.17 29486547 PMC5900369

[B18] La Rovere M. T. Pinna G. D. Maestri R. Mortara A. Capomolla S. Febo O. (2003). Short-term heart rate variability strongly predicts sudden cardiac death in chronic heart failure patients. *Circulation* 107 565–570. 10.1161/01.cir.0000047275.25795.17 12566367

[B19] Littell R. C. Pendergast J. Natarajan R. (2000). Modeling covariance structure in the analysis of repeated measures data. *Stat. Med.* 19 1793–1819. 10.1002/1097-0258(20000715)19:13<1793::aid-sim482>3.3.co;2-h 10861779

[B20] Low J. A. Chan D. K. Y. (2002). Air travel in older people. *Age Ageing* 31 17–22. 10.1093/ageing/31.1.17 11850303

[B21] McNeely E. Spengler J. D. Watson J. (2011). *). Health Effects of Aircraft Cabin pressure in Older and Vulnerable Passengers, Report No. RITE-ACER-CoE-2011–2011.* Boston, MA: Airliner Cabin Environment Research.

[B22] Nable J. V. Tupe C. L. Gehle B. D. Brady W. J. (2015). In-flight medical emergencies during commercial travel. *N. Engl. J. Med.* 373 939–945. 10.1056/nejmra1409213 26332548

[B23] Nesthus T. Wise R. (1997). *Effects of Simulated General Aviation Altitude Hypoxia on Smokers and Non-Smokers.* Washington, DC: Office of Aviation Medicine.

[B24] Neubauer J. A. (2001). Invited review: physiological and pathophysiological responses to intermittent hypoxia. *J. Appl. Physiol.* 90 1593–1599. 10.1152/jappl.2001.90.4.1593 11247965

[B25] Nolan J. Batin P. D. Andrews R. Lindsay S. J. Brooksby P. Mullen M. (1998). Prospective study of heart rate variability and mortality in chronic heart failure: results of the United Kingdom heart failure evaluation and assessment of risk trial (UK-heart). *Circulation* 98 1510–1516. 10.1161/01.cir.98.15.1510 9769304

[B26] Oliveira-Silva I. Leicht A. S. Moraes M. R. Simoes H. G. Del Rosso S. Cordova C. (2016). Heart rate and cardiovascular responses to commercial flights: relationships with physical fitness. *Front. Physiol.* 7:648. 10.3389/fphys.2016.00648 28082914 PMC5186762

[B27] Park S. K. O’Neill M. S. Vokonas P. S. Sparrow D. Schwartz J. (2005). Effects of air pollution on heart rate variability: the VA normative aging study. *Environ. Health Perspect.* 113 304–309. 10.1289/ehp.7447 15743719 PMC1253756

[B28] Perret-Guillaume C. Joly L. Benetos A. (2009). Heart rate as a risk factor for cardiovascular disease. *Prog. Cardiovasc. Dis.* 52 6–10. 10.1016/j.pcad.2009.05.003 19615487

[B29] Peterson D. C. Martin-Gill C. Guyette F. X. Tobias A. Z. McCarthy C. E. Harrington S. T. (2013). Outcomes of medical emergencies on commercial airline flights. *N. Engl. J. Med.* 368 2075–2083. 10.1056/NEJMoa1212052 23718164 PMC3740959

[B30] Pinheiro J. Bates D. DebRoy S. Sarkar D. R Core Team. (2018). *nlme**: Linear and Nonlinear Mixed Effects Models. R package version 3.1-137.* Available at: https://CRAN.R-project.org/package=nlme (accessed January 15, 2019).

[B31] Priebe H. J. (2000). The aged cardiovascular risk patient. *Br. J. Anaesthes.* 85 763–778. 10.1093/bja/85.5.763 11094595

[B32] R Core Team (2018). *R: A Language and Environment for Statistical Computing.* Vienna: R Foundation for Statistical Computing.

[B33] Rodenberg H. (1988). Prevention of medical emergencies during air travel. *Am. Fam. Phys.* 37 263–271. 3344648

[B34] Rosenberg S. R. Kalhan R. Mannino D. M. (2015). Epidemiology of chronic obstructive pulmonary disease: prevalence, morbidity, mortality, and risk factors. *Semin. Respir. Crit. Care Med.* 36 457–469. 10.1055/s-0035-1555607 26238634

[B35] Sauvet F. Jouanin J. C. Langrume C. Van Beers P. Papelier Y. Dussault C. (2009). Heart-rate variability in novice pilots during and after a multi-leg cross-country flight. *Aviat. Space Environ. Med.* 80 862–869. 10.3357/asem.2531.2009 19817238

[B36] Shaffer F. Ginsberg J. P. (2017). An overview of heart rate variability metrics and norms. *Front. Public Health* 5:258. 10.3389/fpubh.2017.00258 29034226 PMC5624990

[B37] Sharma G. Goodwin J. (2006). Effect of aging on respiratory system physiology and immunology. *Clin. Interv. Aging* 1 253–260. 10.2147/ciia.2006.1.3.253 18046878 PMC2695176

[B38] Silverman D. Gendreau M. (2009). Medical issues associated with commercial flights. *Lancet* 373 2067–2077. 10.1016/S0140-6736(09)60209-9 19232708 PMC7137984

[B39] Task Force of the European Society of Cardiology and the North American Society of Pacing, and Electrophysiology (1996). Heart rate variability: standards of measurement, physiological interpretation and clinical use. *Circulation* 93 1043–1065. 10.1161/01.cir.93.5.1043 8598068

[B40] Tsuji H. Larson M. G. Venditti F. J. Jr. Manders E. S. Evans J. C. Feldman C. L. (1996). Impact of reduced heart rate variability on risk for cardiac events. The Framingham Heart Study. *Circulation* 94 2850–2855. 10.1161/01.cir.94.11.2850 8941112

[B41] U.S. Department of Health and Human Services (2004). *The Health Consequences of Smoking: A Report of the Surgeon General.* Washington, DC: U.S. DHHS.

[B42] Vaernes R. J. Owe J. O. Myking O. (1984). Central nervous reactions to a 6.5-hour altitude exposure at 3,048 meters. *Aviat. Space Environ. Med*. 55 921–926. 6497822

[B43] Verjee M. A. Crone R. Ostrovskiy G. (2018). Medical issues in flight and updating the emergency medical kit. *Open Access Emerg. Med.* 10 47–51. 10.2147/OAEM.S152777 29750057 PMC5933470

[B44] Wehrwein E. A. Orer H. S. Barman S. M. (2016). Overview of the anatomy, physiology, and pharmacology of the autonomic nervous system. *Compr. Physiol.* 6 1239–1278. 10.1002/cphy.c150037 27347892

[B45] Wilkins M. R. Ghofrani H. A. Weissman N. Aldashev A. Zhao L. (2015). Pathophysiology and treatment of high-altitude pulmonary vascular disease. *Circulation* 131 582–590. 10.1161/circulationaha.114.006977 25666980

[B46] Winck J. C. Drummond M. Almeida J. Marques J. A. (2008). Air travel and hypoxaemia in real life. *Eur. Respir. J.* 32 236–237. 10.1183/09031936.00001708 18591342

[B47] World Health Organization, Media Center (2004). *World Health Organization Launches New Initiative to Address the Health Needs of a Rapidly Ageing Population.* Available at: http://www.who.int/mediacentre/news/releases/2004/pr60/en/ (accessed July 20, 2011). 15672498

