# Supplementary material for: Changes in Heart Rate and Rhythm During a Crossover Study of Simulated Commercial Flight in Older and Vulnerable Participants
Source: Front Physiol. 2019 Oct 24;10:1339. doi: 10.3389/fphys.2019.01339 (PMC6821722; doi:10.3389/fphys.2019.01339)
Supplement: Supplementary file 1 [file Data_Sheet_1.pdf]

Supplemental File to Changes in Heart Rate and Rhythm During a Crossover Study of Simulated Commercial Flight in Older and Vulnerable Participants

Mark J. Meyer, PhD[1*], Irina Mordukhovich, PhD[2], Gregory A. Wellenius, ScD[3], Murray A. Mittleman, MD, DrPH[4], John P. McCracken, MPH, PhD[5], Brent A. Coull, PhD[6], Eileen McNeely, PhD[2]

[1]Department of Mathematics and Statistics, Georgetown University, Washington, DC, USA

[2]SHINE in the Department of Environmental Health, Harvard T. H. Chan School of Public Health, Boston, MA, USA

[3]Center for Environmental Health and Technology, Brown University, Providence, RI, USA

[4]Department of Epidemiology, Harvard T.H. Chan School of Public Health, Boston, MA, USA

[5]Center for Health Studies, Universidad del Valle de Guatemala, Guatemala City, Guatemala

[6]Department of Biostatistics, Harvard T. H. Chan School of Public Health, Boston, MA, USA

Introduction

This file contains supplementary material referenced in the manuscript "Changes in Heart Rate and Rhythm During a Crossover Study of Simulated Commercial Flight in Older and Vulnerable Participants."

Additional Graphical Results

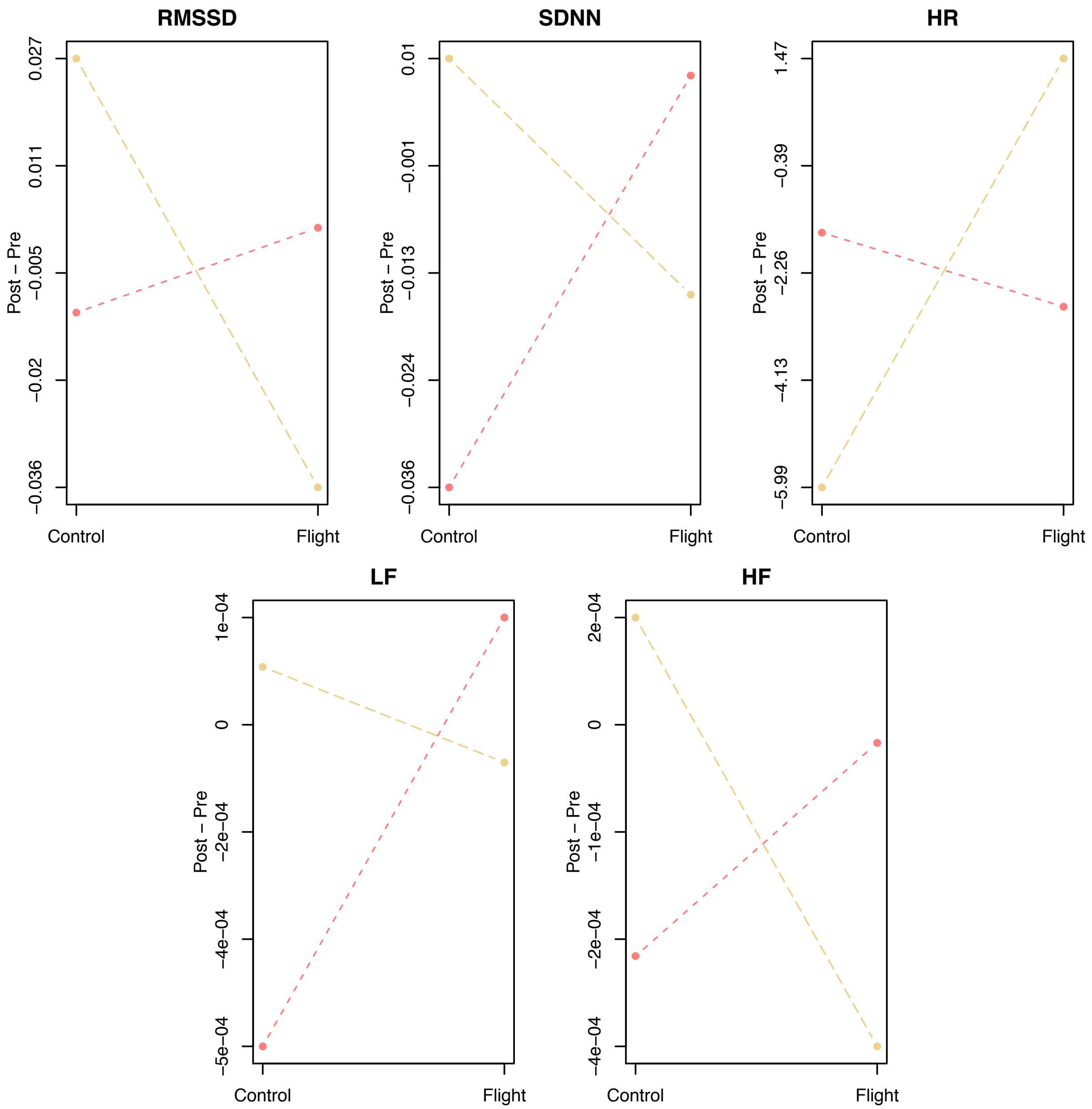

Supplemental Figure 1: Unadjusted, average difference between pre-condition and post for heart rate (HR) and HRV (RMSSD, SDNN, HF, LF) broken down by simulated flight and control day for the two subjects removed as outliers. The differences are first averaged over all available measurements for each subject's control and flight days. Dotted gold lines represent the participant deemed an outlier on Post – Pre changes in RMSSD during both simulated flight and control days. Dashed red lines represent the participant deemed an outlier on Post – Pre changes in SDDN during the control day.

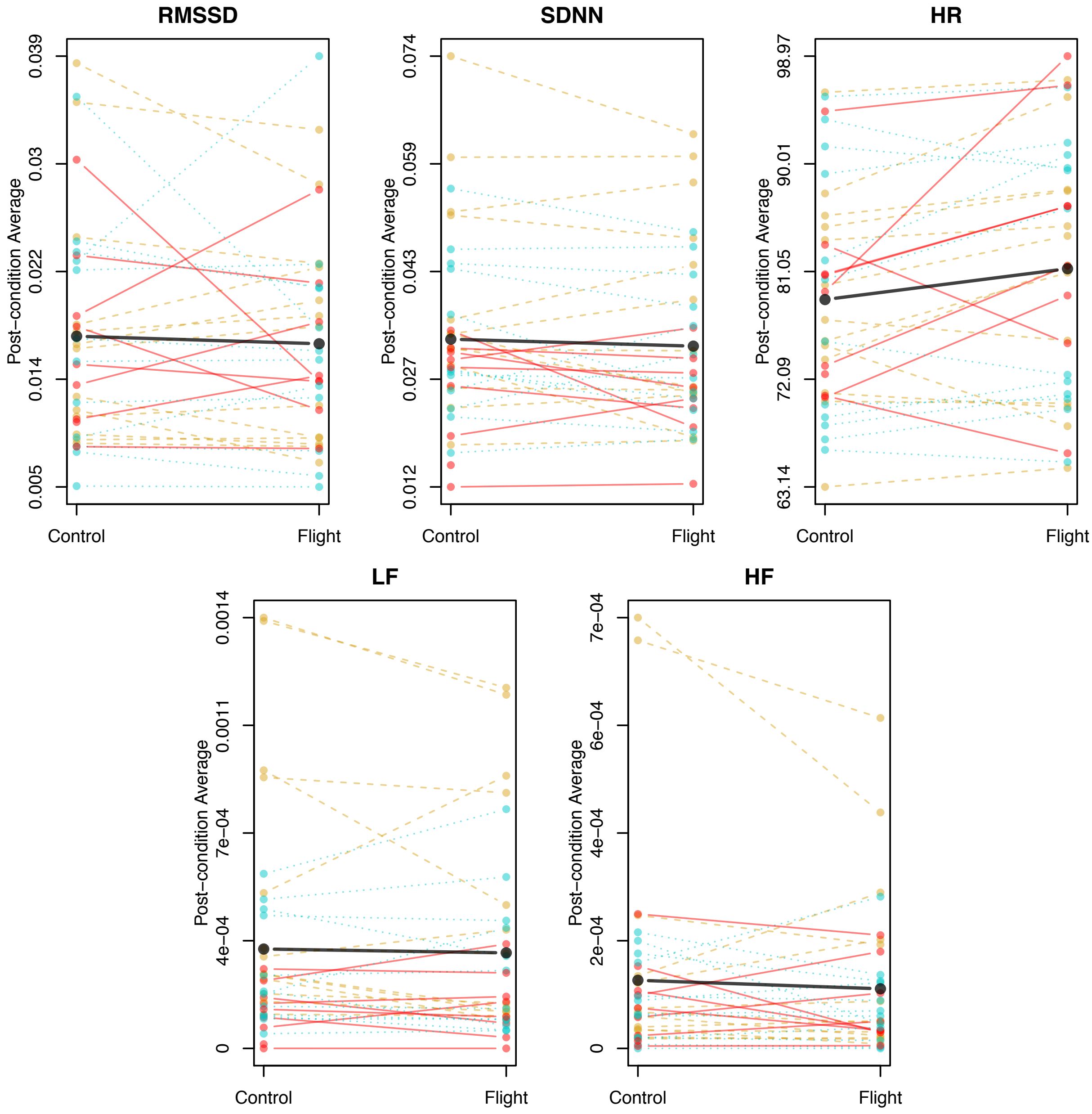

Supplemental Figure 2: Unadjusted, average heart rate and heart rate variability during post-condition broken down by simulated flight and control day for each subject. These values are averaged over all available measurements for each subject's control and flight days. Solid red lines and points denote each Heart Failure participant's mean difference for a given day, dashed gold lines and points denote Healthy participants, and dotted cyan lines and points denote the Smokers. Solid black lines and points denote the mean value across all subject measurements on the given day.

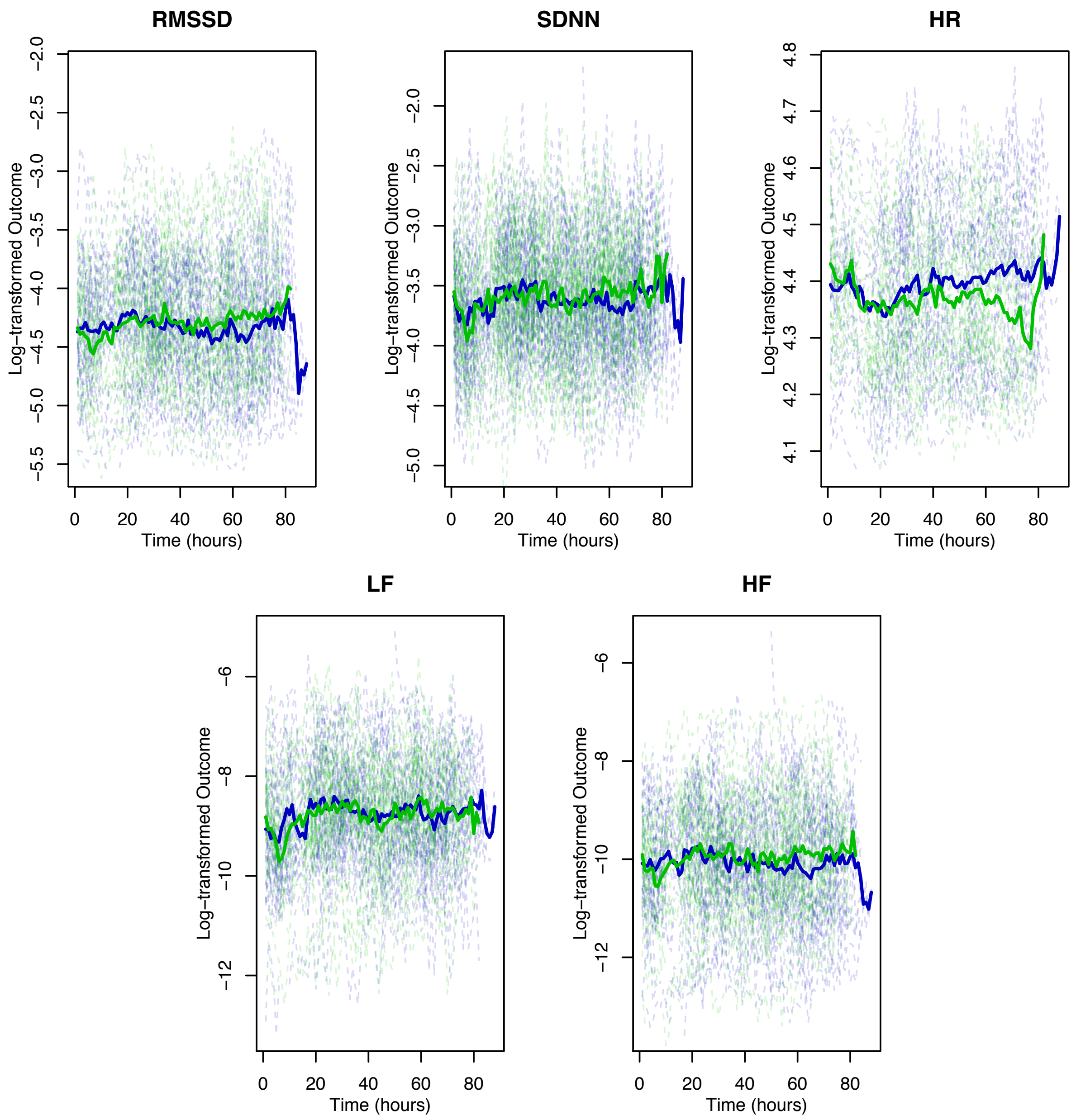

Supplemental Figure 3: Individual and average trajectories for log-transformed heart rate and heart rate variability on both simulated flight and control days. Dashed, translucent lines represent individual trajectories, solid curves represent average trajectories for each day. Measurements from the simulated flight day are in blue while green curves are from control days.

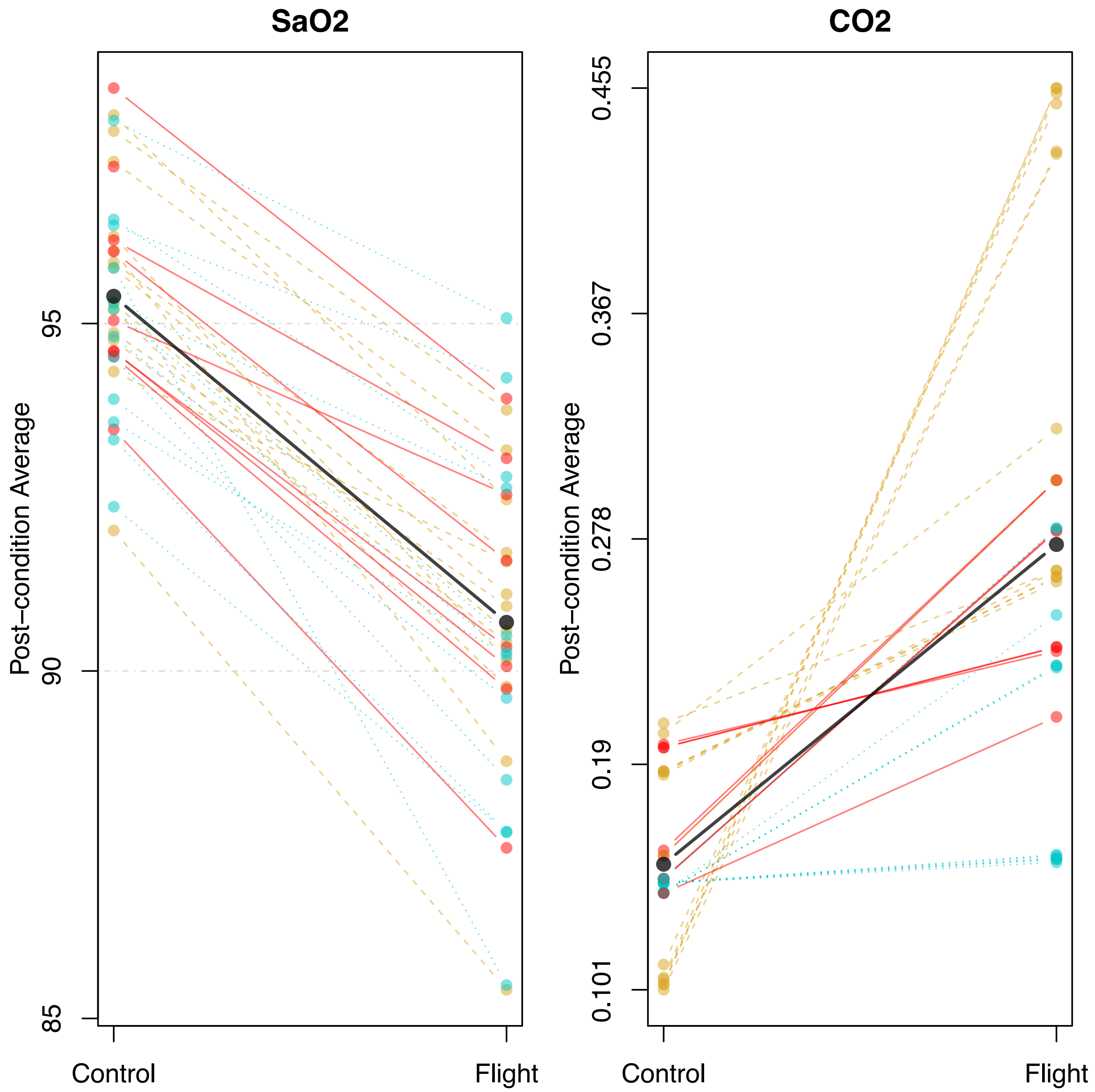

Supplemental Figure 4: Average post-condition SaO2 and CO2 levels broken down by simulated flight and control day for each subject. Solid red lines and points denote each Heart Failure participant's mean difference for a given day, dashed gold lines and points denote Healthy participants, and dotted cyan lines and points denote the Smokers. Solid black lines and points denote the mean value across all subjects on the given day. Dotted-dashed lines at SaO2 equal to 90 and 95 are included for reference.
